# Fractional view analysis of sexual transmitted human papilloma virus infection for public health

**DOI:** 10.1038/s41598-024-53696-8

**Published:** 2024-02-06

**Authors:** Mohammed Cherif Bahi, Salma Bahramand, Rashid Jan, Salah Boulaaras, Hassan Ahmad, Rafik Guefaifia

**Affiliations:** 1 Department of Mathematics and Computer Science, Echahid Cheikh Larbi Tebessi University, Tebessa, Algeria; 2 Laboratory of Mathematics, Informatics and Systems, Echahid Cheikh Larbi Tebessi University, Tebessa, Algeria; 3 Department of Political Science, Bacha Khan University Charsadda, Charsadda, 24420 KPK Pakistan; 4 Department of Civil Engineering, College of Engineering, Institute of Energy Infrastructure (IEI), Universiti Tenaga Nasional (UNITEN), Putrajaya Campus, Jalan IKRAM-UNITEN, 43000 Kajang, Selangor Malaysia; 5 Department of Mathematics, College of Science, Qassim University, Buraydah, 51452 Saudi Arabia; 6 Department of Mathematics, University of Swabi, Swabi, 23561 KPK Pakistan

**Keywords:** Diseases, Endocrinology, Health care, Health occupations, Engineering, Mathematics and computing, Physics

## Abstract

The infection of human papilloma virus (HPV) poses a global public health challenge, particularly in regions with limited access to health care and preventive measures, contributing to health disparities and increased disease burden. In this research work, we present a new model to explore the transmission dynamics of HPV infection, incorporating the impact of vaccination through the Atangana–Baleanu derivative. We establish the positivity and uniqueness of the solution for the proposed model HPV infection. The threshold parameter is determined through the next-generation matrix method, symbolized by ${\mathcal {R}}_0$. Moreover, we investigate the local asymptotic stability of the infection-free steady-state of the system. The existence of the solutions of the recommended model is determined through fixed-point theory. A numerical scheme is presented to visualize the dynamical behavior of the system with variation of input factors. We have shown the impact of input parameters on the dynamics of the system through numerical simulations. The findings of our investigation delineated the principal parameters exerting significant influence for the control and prevention of HPV infection.

## Introduction

Human papilloma virus is a group of related viruses that can infect the genital area, as well as the mouth and throat^1^. It is the most common sexually transmitted infection worldwide. This viral infection is primarily spread through intimate skin-to-skin contact, typically during sexual activity. Notably, there are various types of HPV, and some can cause genital warts, while others are linked to the development of cancers, including cervical, anal, penile, and oropharyngeal cancers^2^. In fact, HPV is a leading cause of cervical cancer in women. Preventive measures include vaccination, which is highly effective in protecting against the most common cancer-causing HPV strains. Regular screening for cervical cancer in women is also essential for early detection and treatment. Given the prevalence of HPV and its potential health implications, education about safe sexual practices, vaccination, and regular medical check-ups are crucial components of public health initiatives aimed at reducing the impact of HPV-related diseases. The HPV vaccine was developed to protect against this viral infection. Given in a series of doses, the vaccination dramatically reduces the incidence of HPV-related malignancies and genital warts^3^. Public health recommendations emphasize the necessity of routine vaccination for adolescents and young adults to improve health outcomes. There is no specific cure for HPV, the infections often clear on their own as the immune system fights the virus^4^. However, certain conditions caused by HPV, such as genital warts or abnormal cervical cells, can be treated. Always consult with a health care professional for personalized advice and treatment options based on your specific situation.

Mathematical models provide a quantitative framework for understanding complex systems and making predictions that can guide actions and policies^5–7^. These modeling typically involves defining variables, formulating equations, and using mathematical tools and techniques to interpret and understand complex phenomena, facilitating problem-solving and decision-making processes in a systematic and quantifiable manner^8–10^. Mathematical models have played a pivotal role in elucidating the dynamics of infectious disease transmission and devising efficacious interventions for disease control^11–13^. Numerous mathematical models have been formulated to conceptualize the key input factors of the dynamics of viral infection within populations^14,15^. Many scientific endeavors have focused on the mathematical modeling of the progression of cervical cancer in conjunction with HPV infection. The objective is to gain a comprehensive understanding of the disease and explore various approaches to managing its advancement or halting it altogether. Several mathematical models have employed a compartmental approach to study the transmission dynamics of HPV infection in the population^16,17^. After that, the researchers in^18,19^ conceptualized and studied the transmission phenomena of cervical cancer by incorporating real parameters from various geographical regions. In^20^, a system comprising five differential equations characterizing HPV, coupled with four compartments delineating epithelial cells healthy, infected, precancerous, and cancerous was delineated. The authors delve into examining the distinct stability of each equilibrium and scrutinize the conditions dictating disease extinction or persistence. Addressing the same issue, a recent study^21^ tackles optimal control by incorporating various incidence functions. The authors propose that the two treatments function by impeding new infections and diminishing the population of precancerous cells. In the present study, we formulate the dynamics of HPV to elucidate the influence of vaccination, asymptomatic carriers, and cervical cancer on the transmission pathway.

In^21,22^, the researchers introduced a saturated infection rate and two viral infection treatments into their model. Biologically, the saturated infection rate signifies the transmission rate of the infection, considering the viral density in the vicinity of the healthy epithelial cell^23,24^. Furthermore, the first treatment functions to gauge the efficacy of drug intervention in preventing new infections, while the second reflects the effectiveness of drug treatment in impeding viral production^25^. In epidemic models, a fractional framework holds significance as it offers a robust mechanism for integrating memory and hereditary property within systems^26,27^. Moreover, non-integer models have the capacity to depict the nonlocal dynamics of biological processes with greater accuracy compared to classical models^28,29^. Utilizing fractional operators in epidemic models yields more precise outcomes for real-world data than traditional integer models^30–32^. The complex biological processes can be accurately characterized through a fractional framework. Therefore, we choose to represent the dynamics of HPV infection within this fractional framework, aiming to offer a more precise depiction of the viral infection’s dynamics.

The structure of the paper unfolds as follows: Section “Theory of fractional-calculus” introduces the fundamental findings and concepts associated with the Atangana–Baleanu fractional operator. In Section “Fractional order model formulation”, we construct a mathematical model for HPV infection, considering vaccination and reinfection phenomena. The stability study is detailed in Section “Analysis of the model”, and Section “Fractional-order model solution” delves into the existence and uniqueness of the solution. Section “Fractional dynamics via Newton polynomial” showcases numerical iterative methods and simulation results. Finally, Section “Conclusion” provides a summary of the entire work.

## Theory of fractional-calculus

In this section, we will unveil pivotal theory of the Atangana–Baleanu operator alongside the classical Caputo derivative, as elucidated in reference^33^. Additionally, we will delve into the Atangana–Baleanu operator, as outlined in reference^33^. These basic concepts and findings will be utilized in the analysis of the model.

### Definition 2.1

^33^. Consider a function *k* such that $k:[p,q]\rightarrow {\mathbb {R}}$, then the Caputo fractional derivative of order $\upsilon$ on *k* can be stated pas

$$\begin{aligned} {}^{{C}}_{p}D^{\upsilon }_{t}(k(t))= \frac{1}{\Gamma (r-\upsilon )}\int _p^t k^r(\varsigma )(t-\varsigma )^{r-\upsilon -1}d\varsigma , \end{aligned}$$

where $$r \in {\textbf{Z}}$$ and $$\upsilon \in (r-1,r)$$.

### Definition 2.2

Suppose a function *k* such that $k\in {\mathcal {H}}^1(p,q)$, $q>p$, and $\upsilon \in [0,1],$ then AB fractional operator in Caputo structure represented by ABC is defined as

$$\begin{aligned} {}^{{ABC}}_{p}D^{\upsilon }_{t}k(t)= \frac{B(\upsilon )}{1-\upsilon }\int _p^t k'(\varsigma )E_\upsilon \bigg [-\upsilon \frac{(t-\varsigma )^\upsilon }{1-\upsilon }\bigg ]d\varsigma . \end{aligned}$$

### Definition 2.3

Integral of AB derivative is represented by ${}^{{ABC}}_{p}I^{\upsilon }_{t}k(t)$ and defined as

$$\begin{aligned} {}^{{ABC}}_{p}I^{\upsilon }_{t}k(t)= \frac{1-\upsilon }{B(\upsilon )}k(t)+\frac{\upsilon }{B(\upsilon )\Gamma (\upsilon )}\int _p^t k(\varsigma ) (t-\varsigma )^{\upsilon -1}d\varsigma . \end{aligned}$$

Since the fractional-order $\upsilon \rightarrow 0$ implies that the initial function can be attained.

### Theorem 2.1

^33^. Consider a function *k* such as $k \in C[p,q]$, then the following holds

$$\begin{aligned} \Vert {}^{{ABC}}_{p}D^{\upsilon }_{t}(k(t))\Vert <\frac{B(\upsilon )}{1-\upsilon }\Vert k(t)\Vert , \;where\; \Vert k(t)\Vert =max_{p\le t\le q}|k(t)|. \end{aligned}$$

Moreover, the Lipschitz holds for the ABC derivative as

$$\begin{aligned} \Vert {}^{{ABC}}_{p}D^{\upsilon }_{t}k_1(t)-{}^{{ABC}}_{p}D^{\upsilon }_{t}k_2(t)\Vert <\mathcal {\varphi }_1 \Vert k_1(t)-k_2(t)\Vert . \end{aligned}$$

### Theorem 2.2

^33^. The following system of fractional differential equation

$$\begin{aligned} {}^{{ABC}}_{p}D^{\upsilon }_{t}k(t)=u(t), \end{aligned}$$

has a unique solution of the form

$$\begin{aligned} {k(t)=\frac{1-\upsilon }{B(\upsilon )}u(t)+\frac{\upsilon }{B(\upsilon )\Gamma (\upsilon )}\int _p^t u(\varsigma )(t-\varsigma )^{\upsilon -1}d\varsigma .} \end{aligned}$$

##  Fractional order model formulation

In this part, we present a mathematical model of HPV transmission. In formulation of the model, we represent the total population by ${\mathcal {N}}(t)$. According to their illness state, the model splits the whole population into six sub-classes: Susceptible ${\mathcal {S}}(t)$, Vaccinated ${\mathcal {V}}(t)$, Asymptomatic ${\mathcal {A}}(t)$, Infected ${\mathcal {I}}(t)$, Recovered ${\mathcal {R}}(t)$, and Cervical cancer ${\mathcal {C}}(t)$. Here, the following presumptions are used to build a mathematical model of the human papilloma virus. A portion $p$ are vaccinated on the onset of an outbreak and a fraction $\alpha$ of the susceptible are vaccinated. After vaccination a portion $\varphi$ of vaccinated individuals moves to susceptible class after losing the effectiveness of the vaccination while the recovered individuals lose the immunity with a rate $\omega$ and become susceptible. The susceptible population is at risk of infection, whether it is from asymptomatic or symptomatic individuals, and this risk is quantified by a force of infection denoted as $\lambda =\frac{\beta [{\mathcal {I}} + \gamma {\mathcal {A}}]}{{\mathcal {N}}}$. Here, $\beta$ is calculated as the product of $\kappa$(the contact rate) and $\tau$(the probability that a contact leads to infection), and $\gamma$ represents the transmission coefficient for asymptomatic individuals. In cases where $\gamma > 1$, asymptomatic individuals are more likely to infect susceptible individuals than symptomatic ones. When $\gamma = 1$, both asymptomatic and symptomatic individuals have an equal chance of infecting the susceptible population. However, if $\gamma < 1$, symptomatic individuals have a greater likelihood of infecting susceptible individuals compared to asymptomatic ones.

**Figure 1:**
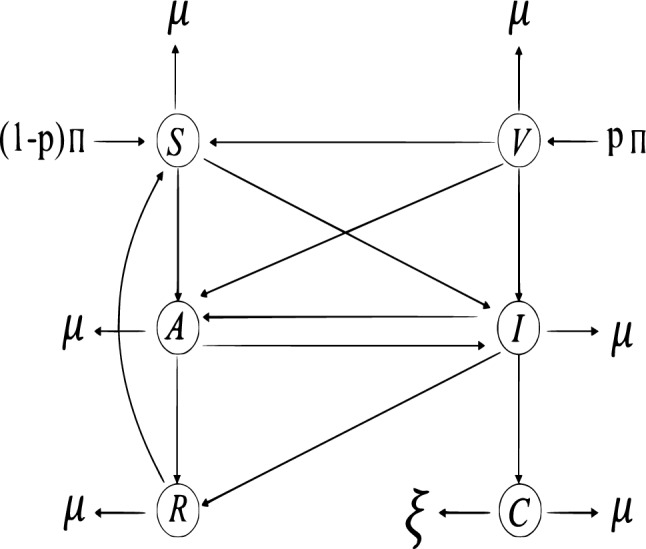
Illustration of the flow chart of the dynamics of the infection of human papilloma virus.

The HPV vaccine is considered to provide only temporary immunity, and individuals who have been vaccinated may still have a chance of being infectious or asymptomatic, although this likelihood is relatively low. The force of infection for the vaccinated population is denoted as $\lambda _v = \varepsilon \lambda$, where $\varepsilon$ falls within the range of 0 to 1. $\varepsilon$ represents the proportion of the serotype not covered by the vaccine. Individuals acquiring new infections due to the force of infection face dual potential outcomes. They can either undergo asymptomatic infection, characterized by a probability denoted as $\rho$, and subsequently join the asymptomatic class. Alternatively, there is a probability of $1-\rho$ that they progress to the infected class. Within the asymptomatic class, individuals encounter two distinct trajectories. They may either manifest disease symptoms or opt for screening, triggering a transition into the infected class at a rate represented as $\theta$. Alternatively, they may naturally recover, acquiring immunity at a rate denoted as $\phi$. Individuals within the infected class undergo transitions based on treatment. At a rate of $\eta$, some individuals move to the recovered compartment through effective treatment, where a proportion $q$ successfully joins the recovered class. Others, constituting the remaining $(1- q )$ proportion, opt for an alternative treatment path, joining the asymptomatic class. Unfortunately, in cases where the treatment fails, individuals may progress to develop cervical cancer at a rate $\delta$, leading to a shift into the cervical cancer compartment. Individuals who are afflicted with cervical cancer may face mortality due to the infection, occurring at a rate denoted as $\xi$. Within all compartments, $\mu$ represents the natural mortality rate of individuals. The flow chart of the transmission dynamics of HPV with the above assumptions is illustrated in Fig. 1. Then, the model of HPV with the above assumptions in the form of mathematical expression is as follows:

1 $$\begin{aligned} \left\{ \begin{array}{rcl} \frac{d{\mathcal {S}}}{dt} &{}=&{} (1 - p )\Pi + \varphi {\mathcal {V}} - ( \rho \lambda + \mu ) {\mathcal {S}} + \omega {\mathcal {R}} \\ \frac{d{\mathcal {V}}}{dt} &{}=&{} p \Pi - (\varphi + \varepsilon \lambda + \mu ){\mathcal {V}} \\ \frac{d{\mathcal {A}}}{dt} &{}=&{} \rho \lambda {\mathcal {S}} + \rho \varepsilon \lambda {\mathcal {V}} + (1 - q )\eta {\mathcal {I}} - (\theta + \phi + \mu ){\mathcal {A}} \\ \frac{d{\mathcal {I}}}{dt} &{}=&{} (1 - \rho )\lambda {\mathcal {S}} + (1 - \rho )\varepsilon \lambda {\mathcal {V}} + \theta {\mathcal {A}} - (\delta + \eta + \mu ) {\mathcal {I}} \\ \frac{d{\mathcal {R}}}{dt} &{}=&{} \phi {\mathcal {A}} + q \eta {\mathcal {I}} - (\omega + \mu ){\mathcal {R}} \\ \frac{d{\mathcal {C}}}{dt} &{}=&{} \delta {\mathcal {I}} - (\xi + \mu ){\mathcal {C}}, \end{array}\right. \end{aligned}$$

with appropriate initial condition

2 $$\begin{aligned} \begin{array}{rcl} {\mathcal {S}}(0)\ge 0,\ {\mathcal {V}}(0)\ge 0,\ {\mathcal {A}}(0)\ge 0,\ {\mathcal {I}}(0)\ge 0,\ {\mathcal {R}}(0)\ge 0,\ {\mathcal {C}}(0)\ge 0. \end{array} \end{aligned}$$

The initial conditions for the system of model (1) are all non-negative and represented as follows: ${\mathcal {S}}(0) = {\mathcal {S}}_0, {\mathcal {V}}(0) = {\mathcal {V}}_0, {\mathcal {A}}(0) = {\mathcal {A}}_0, {\mathcal {I}}(0) = {\mathcal {I}}_0, {\mathcal {R}}(0) = {\mathcal {R}}_0, {\mathcal {C}}(0) = {\mathcal {C}}_0$. The above model (1) in fractional form can written as:

3 $$\begin{aligned} \left\{ \begin{array}{rcl} ^{ABC}_0 D^{\upsilon }_t {\mathcal {S}} &{}=&{} (1 - p )\Pi + \varphi {\mathcal {V}} - ( \rho \lambda + \mu ) {\mathcal {S}} + \omega {\mathcal {R}}, \\ ^{ABC}_0 D^{\upsilon }_t {\mathcal {V}} &{}=&{} p \Pi - (\varphi + \varepsilon \lambda + \mu ){\mathcal {V}},\\ ^{ABC}_0 D^{\upsilon }_t {\mathcal {A}} &{}=&{} \rho \lambda {\mathcal {S}} + \rho \varepsilon \lambda {\mathcal {V}} + (1 - q )\eta {\mathcal {I}} - (\theta + \phi + \mu ){\mathcal {A}}, \\ ^{ABC}_0 D^{\upsilon }_t {\mathcal {I}} &{}=&{} (1 - \rho )\lambda {\mathcal {S}} + (1 - \rho )\varepsilon \lambda {\mathcal {V}} + \theta {\mathcal {A}} - (\delta + \eta + \mu ) {\mathcal {I}}, \\ ^{ABC}_0 D^{\upsilon }_t {\mathcal {R}} &{}=&{} \phi {\mathcal {A}} + q \eta {\mathcal {I}} - (\omega + \mu ){\mathcal {R}}, \\ ^{ABC}_0 D^{\upsilon }_t {\mathcal {C}} &{}=&{} \delta {\mathcal {I}} - (\xi + \mu ){\mathcal {C}}, \end{array}\right. \end{aligned}$$

where $0< \upsilon \le 1.$ The adoption of fractional derivatives in epidemic modeling enhances the models’ ability to reflect the complexity of real-world scenarios, making them more effective tools for predicting and managing the spread of infectious diseases. The Atangana–Baleanu derivative is known for its ability to model non-local and non-singular behaviors, which may be crucial for accurately describing certain physical processes. The Atangana–Baleanu derivative provides a flexible mathematical framework that can be adapted to describe systems with memory and long-range dependencies. Its versatility makes it suitable for a wide range of applications.

### Theorem 3.1

The solutions the system (3) of the disease are nonnegative and bounded for nonnegative initial vales of state variables of the system.

The solutions of our fractional system (3) of the disease is evidently constrained and remains nonnegative for nonnegative initial values of state variables. Consequently, the system is biologically valid. Further analysis of the model will be presented in the upcoming investigation of the system.

## Analysis of the model

In this section of the, we will investigate our model of HPV for disease-free steady-state, reproduction number and local asymptotic stability. Let the disease-free steady-state is denoted by ${\mathcal {E}}_0$ and can be determined by taking the steady-state of system (3) without infection, then, we have

$$\begin{aligned} {\mathcal {E}}_0= ({\mathcal {S}}_0,{\mathcal {V}}_0,0,0,0,0). \end{aligned}$$

Here, we assume that the basic reproduction number is indicated by ${\mathcal {R}}_0$ which can be calculated through different technique. We take the following step to determined ${\mathcal {R}}_0$ of our model:

$$\begin{aligned} F=\begin{bmatrix} &{}\rho \lambda {\mathcal {S}} + \rho \varepsilon \lambda {\mathcal {V}} \\ &{}(1 - \rho )\lambda {\mathcal {S}} + (1 - \rho )\varepsilon \lambda {\mathcal {V}} \\ &{}0 \end{bmatrix}, V=\begin{bmatrix} &{}(\theta + \phi + \mu ){\mathcal {A}} - (1 - q )\eta {\mathcal {I}} \\ &{}(\delta + \eta + \mu ) {\mathcal {I}} - \theta {\mathcal {A}} \\ &{}(\xi + \mu ){\mathcal {C}} - \delta {\mathcal {I}} \end{bmatrix}, \end{aligned}$$

putting $\lambda =\frac{\beta [{\mathcal {I}} + \gamma {\mathcal {A}}]}{{\mathcal {N}}}$, we have

$$\begin{aligned} F=\begin{bmatrix} &{}\frac{\rho \beta [{\mathcal {I}} + \gamma {\mathcal {A}}]{\mathcal {S}}}{{\mathcal {N}}} + \frac{\rho \varepsilon \beta [{\mathcal {I}} + \gamma {\mathcal {A}}]{\mathcal {V}}}{{\mathcal {N}}} \\ &{}\frac{(1 - \rho )\beta [{\mathcal {I}} + \gamma {\mathcal {A}}] {\mathcal {S}}}{{\mathcal {N}}} + \frac{(1 - \rho )\varepsilon \beta [{\mathcal {I}} + \gamma {\mathcal {A}}]{\mathcal {V}}}{{\mathcal {N}}} \\ &{}0 \end{bmatrix}, V=\begin{bmatrix} &{}(\theta + \phi + \mu ){\mathcal {A}} - (1 - q )\eta {\mathcal {I}} \\ &{}(\delta + \eta + \mu ) {\mathcal {I}} - \theta {\mathcal {A}} \\ &{}(\xi + \mu ){\mathcal {C}} - \delta {\mathcal {I}} \end{bmatrix}. \end{aligned}$$

Taking the Jacobian of the above, we have ${\mathcal {F}}$ and ${\mathcal {V}}$ as given below

$$\begin{aligned} {\mathcal {F}}=\begin{bmatrix} \rho \beta \gamma k_1 &{}\rho \beta k_1 &{}0 \\ (1-\rho )\beta \gamma k_1 &{}(1-\rho )\beta k_1 &{}0 \\ 0 &{}0 &{}0 \end{bmatrix}, \ \ and \ \ {\mathcal {V}}=\begin{bmatrix} c &{}-(1- q )\eta &{}0 \\ -\theta &{} d &{}0 \\ 0 &{}-\delta &{} f \end{bmatrix}, \end{aligned}$$

in which $q_1 = \frac{\varphi - \mu + \mu p}{\varphi + \mu }$, $q_2 = \frac{ \mu p}{ \varphi + \mu }$, $k_1 = q_1 + \varepsilon q_2$, $k_2 = cd - \theta \eta (1 - q)$, $k_3 = d\gamma + \theta$, $k_4 = (1 - q)\eta \gamma + c$, $a = ( \lambda + \mu )$, $b = (\varphi + \epsilon \lambda + \mu )$, $c = (\theta + \varphi + \mu )$, $d = (\delta + \eta + \mu )$, $e = (\omega + \mu )$ and $f = (\xi + \mu )$. From the above, we have ${\mathcal {F}} {\mathcal {V}}^{-1}$ as

$$\begin{aligned} {\mathcal {F}} {\mathcal {V}}^{-1}= & {} \begin{bmatrix} \rho \beta \gamma k_1 &{}\rho \beta k_1 &{}0 \\ (1-\rho )\beta \gamma k_1 &{}(1-\rho )\beta k_1 &{}0 \\ 0 &{}0 &{}0 \end{bmatrix}\begin{bmatrix} \frac{d}{k_2} &{}\frac{(1-q)\eta }{k_2} &{}0 \\ \frac{\theta }{k_2} &{}\frac{c}{k_2} &{}0 \\ \frac{-\theta \delta }{f k_2} &{}\frac{c\delta }{f k_2} &{}\frac{1}{f} \end{bmatrix} \\= & {} \begin{bmatrix} \frac{\rho \beta k_1 k_3}{k_2} + \frac{\rho \beta k_1}{k_2} &{}\frac{(1-q)\eta \rho \beta \gamma k_1}{k_2} + \frac{c\rho \beta k_1 }{k_2} &{}0 \\ \frac{d\rho \beta \gamma k_1}{k_2} + \frac{\theta \rho \beta k_1}{k_2} &{}\frac{(1-\rho )(1-q)\eta \beta \gamma k_1}{k_2} + \frac{(1-\rho )c\beta k_1}{k_2} &{}0 \\ 0 &{}0 &{}0 \end{bmatrix} \\= & {} \begin{bmatrix} \frac{\rho \beta k_1 k_3}{k_2} &{}\frac{\rho \beta k_1 k_4}{k_2} &{}0 \\ \frac{(1-\rho ) k_1 k_3}{k_2} &{}\frac{(1-\rho )\beta k_1 k_4}{k_2} &{}0 \\ 0 &{}0 &{}0 \end{bmatrix}. \end{aligned}$$

Here, assume that the greatest eigenvalue of ${\mathcal {F}} {\mathcal {V}}^{-1}$ is denoted by $\rho ({\mathcal {F}} {\mathcal {V}}^{-1})$ which is $\frac{\kappa \tau k_1 k_2 k_3\rho + (1 - \rho )k_4}{k_2}$ and the basic reproduction number through next-generation matrix method is the greatest eigenvalue of ${\mathcal {F}} {\mathcal {V}}^{-1}$, thus, we have

$$\begin{aligned} {\mathcal {R}}_0 = \frac{\kappa \tau k_1 k_2 k_3\rho + (1 - \rho )k_4}{k_2}. \end{aligned}$$

### Theorem 4.1

If ${\mathcal {R}}_0<1$, then the steady-state ${\mathcal {E}}_0$ is locally asymptotically stable and is unstable in other cases.

### Proof

For the required stability result, we take the the Jacobian matrix at ${\mathcal {E}}_0$ as

$$\begin{aligned} {\mathcal {J}}(E_0)=\begin{bmatrix} -\mu &{}\varphi &{}\beta \gamma q_1 &{}\beta q_1 &{}\omega &{}0 \\ 0 &{}-b &{}\beta \varepsilon \gamma q_2 &{}\beta \varepsilon q_2 &{}0 &{}0 \\ 0 &{}0 &{}\rho \beta \gamma k_1 - c &{}\rho \beta k_1-(1-q)\eta &{}0 &{}0 \\ 0 &{}0 &{}(1-\rho )\beta \gamma k_1+\theta &{}(1-\rho )\beta k_1-d &{}0 &{}0 \\ 0 &{}0 &{}\phi &{}q\eta &{}-e &{}0 \\ 0 &{}0 &{}0 &{}\delta &{}0 &{}-f \end{bmatrix}. \end{aligned}$$

For the required result, we will show that all the eigenvalues of ${\mathcal {J}}(E_0)$ are negative. For which, we take the characteristic equation $\det [{\mathcal {J}}(E_0) - \chi I] = 0$ as:

$$\begin{aligned} \begin{vmatrix} -\mu -\chi&\varphi&\beta \gamma q_1&\beta q_1&\omega&0 \\ 0&-b-\chi&\beta \varepsilon \gamma q_2&\beta \varepsilon q_2&0&0 \\ 0&0&[\rho \beta \gamma k_1 - c]-\chi&\rho \beta k_1-(1-q)\eta&0&0 \\ 0&0&(1-\rho )\beta \gamma k_1+\theta&[(1-\rho )\beta k_1-d]-\chi&0&0 \\ 0&0&\phi&q\eta&-e-\chi&0 \\ 0&0&0&\delta&0&-f-\chi \end{vmatrix}=0. \end{aligned}$$

From the above, the first and second eigenvalue are $-\mu$ and $-f$ which are negative while the other eigenvalues can be determined from

$$\begin{aligned} \begin{vmatrix} -b-\chi&\beta \varepsilon \gamma q_2&\beta \varepsilon q_2&0 \\ 0&[\rho \beta \gamma k_1 - c]-\chi&\rho \beta k_1-(1-q)\eta&0 \\ 0&(1-\rho )\beta \gamma k_1+\theta&[(1-\rho )\beta k_1-d]-\chi&0 \\ 0&\phi&q\eta&-e-\chi \end{vmatrix}=0, \end{aligned}$$

here, we have the third and fourth eigenvalue are $-b$ and $-e$ which are negative. The remaining eigenvalues can be calculated from

$$\begin{aligned} {\mathcal {J}}(E_1)=\begin{bmatrix} \rho \beta \gamma k_1 - c &{}\rho \beta k_1-(1-q)\eta \\ (1-\rho )\beta \gamma k_1+\theta &{}(1-\rho )\beta k_1-d \end{bmatrix}. \end{aligned}$$

Here, if $det({\mathcal {J}}(E_1))<0$ and $trc({\mathcal {J}}(E_1))>0$ for ${\mathcal {R}}_0<1$, then the disease-free steady-state of our model of HPV is locally asymptotically stable. 

## Fractional-order model solution

In this section of the manuscript, we will utilize fixed-point theory to confirm the uniqueness and existence of solutions of our model of the disease. The described system for HPV with the Atangana-Baleno derivative is provided as follows

4 $$\begin{aligned} \left\{ \begin{array}{rcl} ^{ABC}_0 D^{\upsilon }_t {\mathcal {S}} &{}=&{} (1 - p )\Pi + \varphi {\mathcal {V}} - ( \rho \lambda + \mu ) {\mathcal {S}} + \omega {\mathcal {R}}, \\ ^{ABC}_0 D^{\upsilon }_t {\mathcal {V}} &{}=&{} p \Pi - (\varphi + \varepsilon \lambda + \mu ){\mathcal {V}},\\ ^{ABC}_0 D^{\upsilon }_t {\mathcal {A}} &{}=&{} \rho \lambda {\mathcal {S}} + \rho \varepsilon \lambda {\mathcal {V}} + (1 - q )\eta {\mathcal {I}} - (\theta + \phi + \mu ){\mathcal {A}}, \\ ^{ABC}_0 D^{\upsilon }_t {\mathcal {I}} &{}=&{} (1 - \rho )\lambda {\mathcal {S}} + (1 - \rho )\varepsilon \lambda {\mathcal {V}} + \theta {\mathcal {A}} - (\delta + \eta + \mu ) {\mathcal {I}}, \\ ^{ABC}_0 D^{\upsilon }_t {\mathcal {R}} &{}=&{} \phi {\mathcal {A}} + q \eta {\mathcal {I}} - (\omega + \mu ){\mathcal {R}}, \\ ^{ABC}_0 D^{\upsilon }_t {\mathcal {C}} &{}=&{} \delta {\mathcal {I}} - (\xi + \mu ){\mathcal {C}}, \end{array}\right. \end{aligned}$$

this can be further expressed as follows:

5 $$\begin{aligned} \begin{array}{ll} {}^{{ABC}}_{0} D^{\upsilon }_{t}w(t)={\mathcal {J}}(t,w(t)),\\ w(0)=w_0, \;\; 0<t<{\mathcal {T}}<\infty . \end{array} \end{aligned}$$

In this context, we have the state variables represented by $w(t)=({\mathcal {S}},{\mathcal {V}},{\mathcal {A}},{\mathcal {I}},{\mathcal {R}},{\mathcal {C}})$, and ${\mathcal {J}}$ is a continuous function. To clarify, the vector function ${\mathcal {J}}$ can be more clearly expressed as follows:

$$\begin{aligned} {\mathcal {J}}=\left( \begin{array}{cccccc} {\mathcal {J}}_1\\ {\mathcal {J}}_2\\ {\mathcal {J}}_3\\ {\mathcal {J}}_4\\ {\mathcal {J}}_5\\ {\mathcal {J}}_6 \end{array} \right) =\left( \begin{array}{cccccc} (1 - p )\Pi + \varphi {\mathcal {V}} - ( \rho \lambda + \mu ) {\mathcal {S}} + \omega {\mathcal {R}} \\ p \Pi - (\varphi + \varepsilon \lambda + \mu ){\mathcal {V}} \\ \rho \lambda {\mathcal {S}} + \rho \varepsilon \lambda {\mathcal {V}} + (1 - q )\eta {\mathcal {I}} - (\theta + \phi + \mu ){\mathcal {A}} \\ (1 - \rho )\lambda {\mathcal {S}} + (1 - \rho )\varepsilon \lambda {\mathcal {V}} + \theta {\mathcal {A}} - (\delta + \eta + \mu ) {\mathcal {I}} \\ \phi {\mathcal {A}} + q \eta {\mathcal {I}} - (\omega + \mu ){\mathcal {R}} \\ \delta {\mathcal {I}} - (\xi + \mu ){\mathcal {C}} \end{array} \right) , \end{aligned}$$

with appropriate initial conditions specified as $w_0(t)=({\mathcal {S}}(0),{\mathcal {V}}(0),{\mathcal {A}}(0),{\mathcal {I}}(0),{\mathcal {R}}(0),{\mathcal {C}}(0))$, and furthermore, the function ${\mathcal {J}}$ satisfies the Lipschitz condition as outlined below:

6 $$\begin{aligned} \Vert {\mathcal {J}}(t, l_1(t))-{\mathcal {J}}(t, l_2(t))\Vert\le & {} \textrm{W}\Vert w_1(t)-w_2(t)\Vert . \end{aligned}$$

Subsequently, we will explore the uniqueness and existence of system (4) in the following outcome.

### Theorem 5.1

A unique solution for the suggested system (4) of HPV is present if the following condition is met

7 $$\begin{aligned} \frac{(1-\upsilon )}{ABC(\upsilon )}{\mathcal {V}}+\frac{\upsilon }{ABC(\upsilon )\Gamma (\upsilon )}{\mathcal {T}}^{\upsilon }_{max}{\mathcal {V}}<1. \end{aligned}$$

### Proof

To establish the intended result, we apply the AB fractional integral (2.3) to the system (5) which provide the following

8 $$\begin{aligned} w(t)=w_0+\frac{1-\upsilon }{ABC(\upsilon )}{\mathcal {J}}(t,w(t))+\frac{\upsilon }{ABC(\upsilon )\Gamma (\upsilon )}\int _0^t(t-\varpi )^{\upsilon -1}{\mathcal {J}} (\varpi ,w(\varpi ))d\varpi . \end{aligned}$$

Consider the interval $(0, {\mathcal {T}})$ represented as *I*, and the operator $\Lambda :{\mathcal {P}}(I,\textrm{R}^6)\rightarrow {\mathcal {P}}(I,\textrm{R}^6)$ is defined as follows:

9 $$\begin{aligned} \Lambda [w(t)]=w_0+\frac{1-\upsilon }{ABC(\upsilon )}{\mathcal {J}}(t,w(t))+\frac{\upsilon }{ABC(\upsilon )\Gamma (\upsilon )}\int _0^t(t-\varpi )^{\upsilon -1}{\mathcal {J}} (\varpi ,w(\varpi ))d\varpi . \end{aligned}$$

Then, Eq. (8) can be written as

10 $$\begin{aligned} w(t)=\Lambda [w(t)], \end{aligned}$$

the supremum norm over the set *I* is represented by $\Vert .\Vert _I$, and defined as

11 $$\begin{aligned} \Vert w(t)\Vert _I=\sup _{t\in I}\Vert w(t)\Vert ,\;\;w(t)\in {\mathcal {P}}. \end{aligned}$$

One can observe with patience that ${\mathcal {P}}(I,\textrm{R}^6)$ forms a Banach space equipped with the norm $\Vert .\Vert _I$. Moreover, it is evident that

12 $$\begin{aligned} \bigg \Vert \int _0^t {\mathcal {K}} (t,\varpi )w(\varpi )d\varpi \bigg \Vert \le {\mathcal {T}}\Vert {\mathcal {K}} (t,\varpi )\Vert _I \Vert w(t)\Vert _I, \end{aligned}$$

both *w*(*t*) and ${\mathcal {K}}(t,\varpi )$ are members of ${\mathcal {P}}(I,\textrm{R}^6)$ and ${\mathcal {P}}(I^2,\textrm{R})$, respectively, in a way that

13 $$\begin{aligned} \Vert {\mathcal {K}}(t,\varpi )\Vert _I=\sup _{t,\varpi \in I}|{\mathcal {K}}(t,\varpi )|. \end{aligned}$$

Using the definition of $\Lambda$ as outlined in (10), we obtain the following result

14 $$\begin{aligned} \Vert \Lambda [w_1(t)]- \Lambda [w_2(t)]\Vert _I\le & {} \bigg \Vert \frac{(1-\upsilon )}{ABC(\upsilon )} ({\mathcal {J}} (t,w_1(t))-{\mathcal {J}}(t,w_2(t))+ \frac{\upsilon }{ABC(\upsilon )\Gamma (\upsilon )} \nonumber \\{} & {} \times \int ^t_0(t-\varpi )^{\upsilon -1}({\mathcal {J}}(\varpi ,w_1(\varpi ))- {\mathcal {J}}(\varpi ,w_2(\varpi )))d\varpi \bigg \Vert _I. \end{aligned}$$

Furthermore, by making use of the Lipschitz condition (6) and the outcome from (12), the following is derived

15 $$\begin{aligned} \Vert \Lambda [w_1(t)]- \Lambda [w_1(t)]\Vert _I\le & {} \bigg [\frac{(1-\upsilon ){\mathcal {V}}}{ABC(\upsilon )}+\frac{\upsilon }{ABC(\upsilon )\Gamma (\upsilon )} {\mathcal {V}}{\mathcal {T}}^{\upsilon }_{max}\bigg ]\Vert w_1(t)-w_2(t)\Vert _I. \end{aligned}$$

As a result, we get the following

16 $$\begin{aligned} \Vert \Lambda [w_1(t)]-\Lambda [w_1(t)]\Vert _I\le & {} \textrm{D}\Vert w_1(t)-w_2(t)\Vert _I, \end{aligned}$$

where

$$\begin{aligned} \textrm{D}=\frac{(1-\upsilon ){\mathcal {V}}}{ABC(\upsilon )}+\frac{\upsilon }{ABC(\upsilon )\Gamma (\upsilon )}{\mathcal {V}}{\mathcal {T}}^{\upsilon }_{max}. \end{aligned}$$

It is clear that if condition (7) holds, then $\Lambda$ is a contraction. This, in turn, implies that the HPV system (4) possesses a unique solution. 

**Figure 2:**
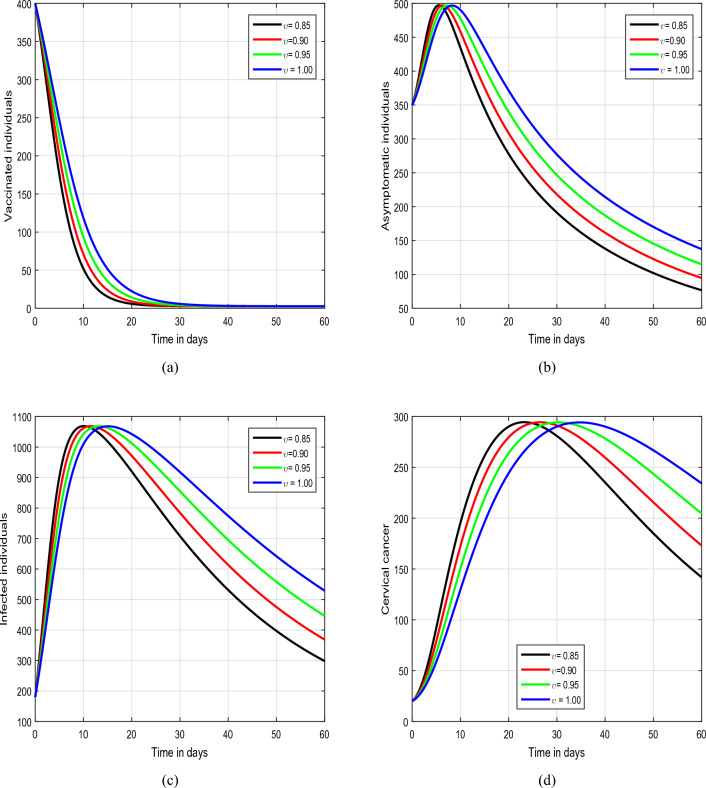
Time series analysis of the proposed model of HPV with the variation of the input factor $\upsilon$, i.e., $\upsilon =0.85, 0.90, 0.95, 1.00$.

**Figure 3:**
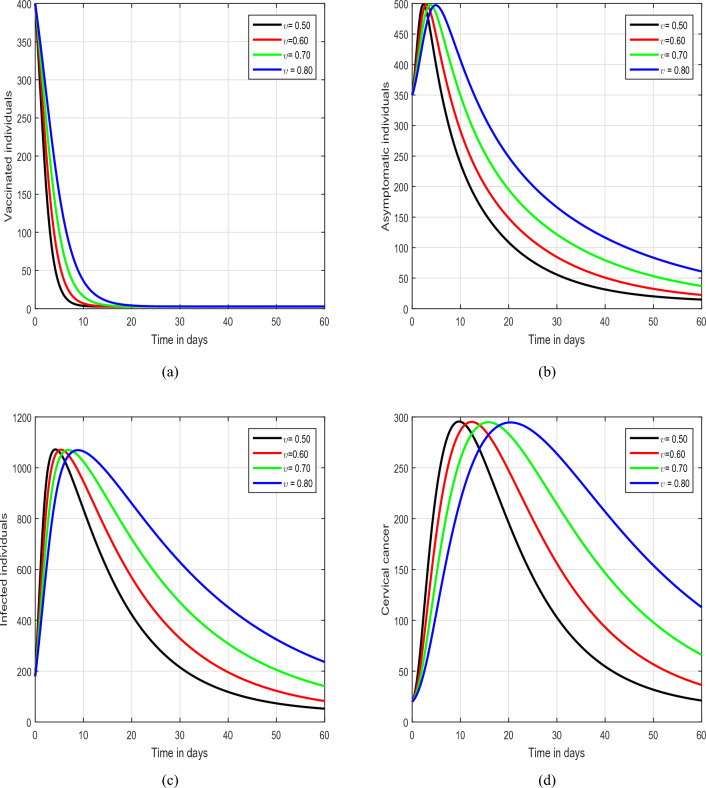
Time series analysis of the proposed model of HPV with the variation of the input factor $\upsilon$, i.e., $\upsilon =0.5, 0.6, 0.7, 0.8$.

**Figure 4:**
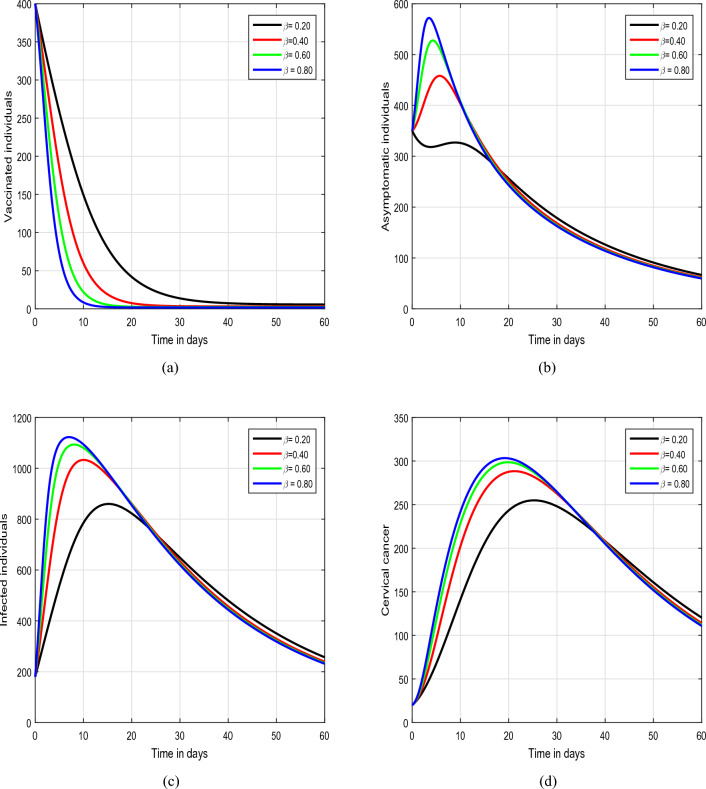
Graphical view analysis of the dynamical behaviour of our model of HPV with different values of input factor $\beta$, i.e., $\beta =0.20, 0.40, 0.60, 0.80$.

**Figure 5:**
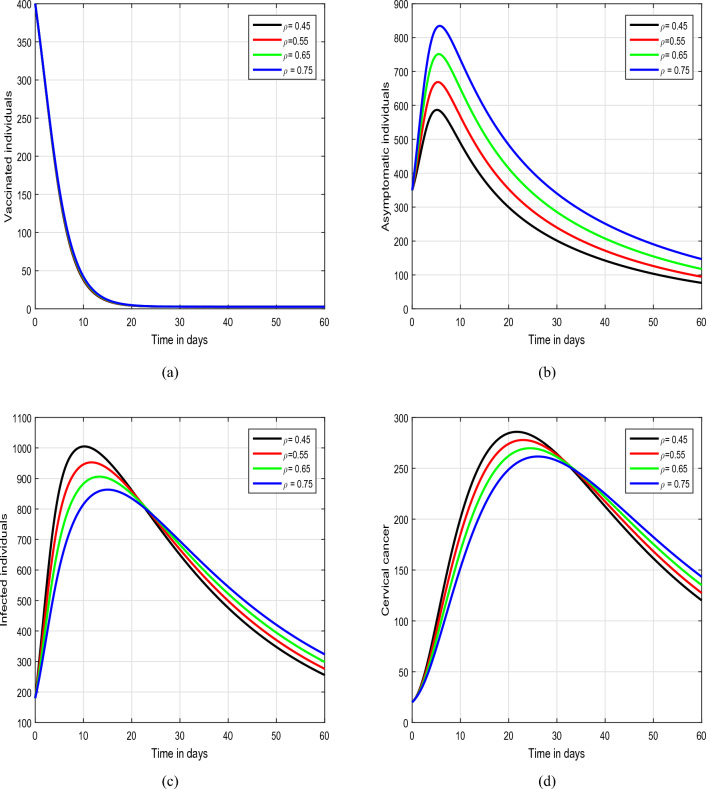
Graphical view analysis of the dynamical behaviour of our model of HPV with different values of input factor $\rho$, i.e., $\beta =0.45, 0.55, 0.65, 0.75$.

**Figure 6:**
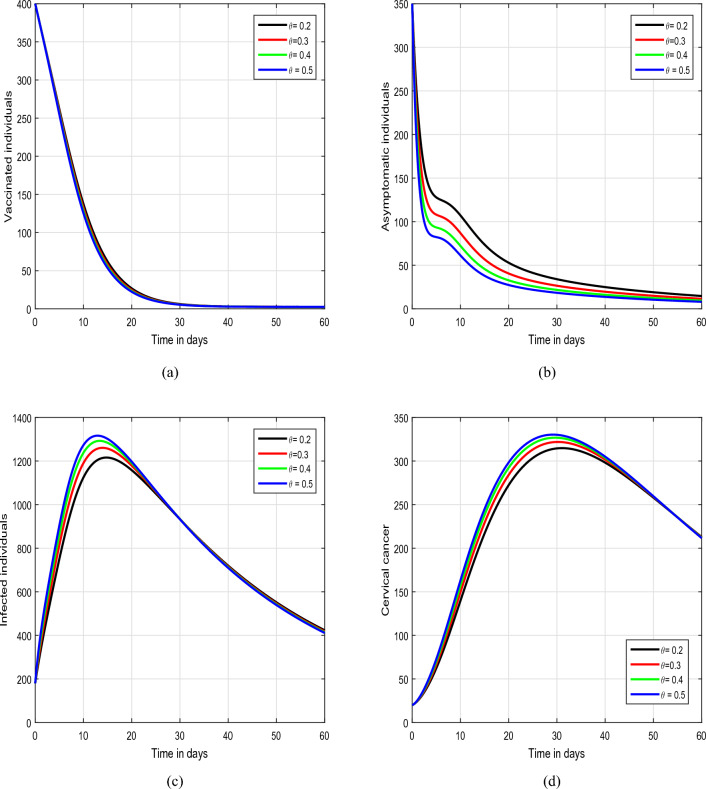
Illustration of the tracking paths of the suggested model of HPV with the variation of the input parameter $\theta$, i.e., $\theta =0.20, 0.30, 0.40, 0.50$.

**Figure 7:**
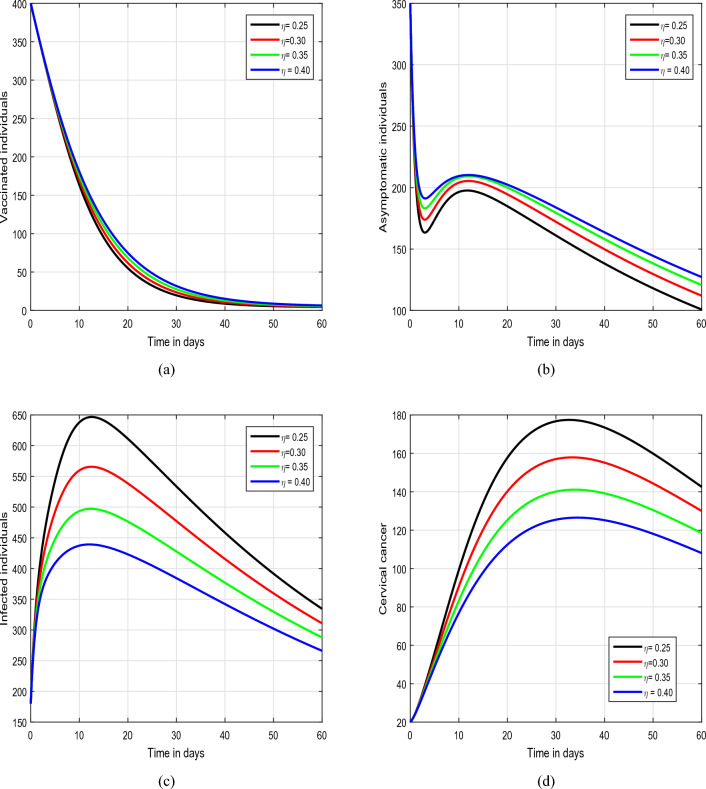
Illustration of the tracking paths of the suggested model of HPV with the variation of the input parameter $\eta$, i.e., $\eta =0.25, 0.30, 0.35, 0.40$.

## Fractional dynamics via Newton polynomial

In this section of the paper, our focus is on the numerical solution of our system (4) of the infection. To do this, we consider the below stated Atangana–Baleanu derivative system

17 $$\begin{aligned} ^{ABC}_0 D^{\upsilon }_t g(t)= f(t,g(t)), \end{aligned}$$

transform the previously stated equation into the subsequent form according to^34^:

18 $$\begin{aligned} g(t)-g(0)= \frac{1- \upsilon }{AB(\upsilon )} f(t,g(t))+ \frac{\upsilon }{AB(\upsilon ) \Gamma (\upsilon )} \int _0^t f(\theta ,g(\theta )) (t-\theta )^{(\upsilon -1)} d\theta , \end{aligned}$$

the above at $t_{r+1}=(r+1)\Delta t$ can be stated as

19 $$\begin{aligned} g(t_{r+1})-g(0)= \frac{1- \upsilon }{AB(\upsilon )} f(t_r,g(t_r))+ \frac{\upsilon }{AB(\upsilon ) \Gamma (\upsilon )} \int _0^{t_{r+1}} f(\theta ,g(\theta )) (t_{r+1}-\theta )^{(\upsilon -1)} d\theta , \end{aligned}$$

this can be further transformed into:

20 $$\begin{aligned} g(t_{r+1})= g(0)+ \frac{1- \upsilon }{AB(\upsilon )} f(t_r,g(t_r))+ \frac{\upsilon }{AB(\upsilon ) \Gamma (\upsilon )} \Sigma _{\imath =2}^{r} \int _{t_\imath }^{t_{\imath +1}} f(\theta ,g(\theta )) d\theta . \end{aligned}$$

In the subsequent phase, we employ the Newton polynomial method to estimate *f*(*t*, *g*(*t*)) as follows

21 $$\begin{aligned} P_r (\theta )= & {} f(t_{r-2},g(t_{r-2}))+ \frac{f(t_{r-1},g(t_{r-1}))-f(t_{r-2},g(t_{r-2}))}{\Delta t} (\theta -t_{r-2})\nonumber \\{} & {} + \frac{f(t_{r},g(t_{r}))- 2 f(t_{r-1},g(t_{r-1}))+f(t_{r-2},g(t_{r-2}))}{2(\Delta t)^2} \times (\theta -t_{r-2})(\theta -t_{r-1}). \end{aligned}$$

Utilizing the above stated polynomial in (20), we get that

22 $$\begin{aligned} g^{r+1}= & {} g^0+ \frac{1- \upsilon }{AB(\upsilon )} f(t_r,g(t_r))\nonumber \\{} & {} + \frac{\upsilon }{AB(\upsilon ) \Gamma (\upsilon )} \sum _{\imath =2}^{r} \int _{t_\imath }^{t_{\imath +1}} \bigg ( f(t_{\imath -2},g^{\imath -2}) \nonumber \\{} & {} + \frac{f(t_{\imath -1},g^{\imath -1})-f(t_{\imath -2},g^{\imath -2})}{\Delta t} (\theta -t_{\imath -2})\nonumber \\{} & {} + \frac{f(t_{\imath },g^{\imath })-2f(t_{\imath -1},g^{\imath -1})+f(t_{\imath -2},g^{\imath -2})}{2 (\Delta t)^2} (\theta -t_{\imath -2})(\theta -t_{\imath -1}) \bigg ) (t_{r+1}-\theta )^{\upsilon -1} d\theta . \end{aligned}$$

Moreover, we get

23 $$\begin{aligned} g^{r+1}= & {} g^0+ \frac{1- \upsilon }{AB(\upsilon )} f(t_r,g(t_r))\nonumber \\{} & {} + \frac{\upsilon }{AB(\upsilon ) \Gamma (\upsilon )} \sum _{\imath =2}^{r} \bigg (\int _{t_\imath }^{t_{\imath +1}} f(t_{\imath -2},g^{\imath -2}) (t_{r+1}-\theta )^{\upsilon -1} d\theta \nonumber \\{} & {} + \int _{t_\imath }^{t_{\imath +1}} \frac{f(t_{\imath -1},g^{\imath -1})-f(t_{\imath -2},g^{\imath -2})}{\Delta t} (\theta -t_{\imath -2}) (t_{r+1}-\theta )^{\upsilon -1} d\theta \nonumber \\{} & {} + \int _{t_\imath }^{t_{\imath +1}} \frac{f(t_{\imath },g^{\imath })-2f(t_{\imath -1},g^{\imath -1})+f(t_{\imath -2},g^{\imath -2})}{2 (\Delta t)^2} (\theta -t_{\imath -2})(\theta -t_{\imath -1}) (t_{r+1}-\theta )^{\upsilon -1} d\theta \bigg ), \end{aligned}$$

the following result is achieved after simplification

24 $$\begin{aligned} g^{r+1}= & {} g^0+ \frac{1-\upsilon }{AB(\upsilon )}f(t_r,y(t_r))\nonumber \\{} & {} + \frac{\upsilon }{AB(\upsilon ) \Gamma (\upsilon )} \sum ^{r}_{\imath =2} f (t_{\imath -2},g^{\imath -2}) \Delta t \int _{t_\imath }^{t_{\imath +1}} (t_{r+1}-\theta )^{\upsilon -1} d\theta \nonumber \\{} & {} +\frac{\upsilon }{AB(\upsilon ) \Gamma (\upsilon )} \sum ^{r}_{\imath =2} \frac{f (t_{\imath -1},g^{\imath -1})-f (t_{\imath -2},g^{\imath -2})}{\Delta t} \int _{t_\imath }^{t_{\imath +1}} (\theta -t_{\imath -2}) (t_{r+1}-\theta )^{\upsilon -1} d\theta \nonumber \\{} & {} +\frac{1}{\Gamma (\upsilon )} \sum ^{r}_{\imath =2} \frac{f (t_{\imath },g^{\imath })-2f (t_{\imath -1},g^{\imath -1})+f (t_{\imath -2},g^{\imath -2})}{2 (\Delta t)^2} \nonumber \\{} & {} \times \int _{t_\imath }^{t_{\imath +1}} (\theta -t_{\imath -2})(\theta -t_{\imath -1}) (t_{r+1}-\theta )^{\upsilon -1} d \theta , \end{aligned}$$

the integrals above can be evaluated using the following method

25 $$\begin{aligned} \int _{t_\imath }^{t_{\imath +1}} (t_{r+1}-\theta )^{\upsilon -1} d \theta= & {} \frac{(\Delta t)^\upsilon }{\upsilon } \bigg ( (r-\imath +1)^\upsilon -(r-\imath )^\upsilon \bigg ) \nonumber \\ \int _{t_\imath }^{t_{\imath +1}} (\theta -t_{\imath -2}) (t_{r+1}-\theta )^{\upsilon -1} d \theta= & {} \frac{(\Delta t)^{\upsilon +1}}{\upsilon (\upsilon +1)} \bigg ( (r-\imath +1)^\upsilon (r-\imath +3+2 \upsilon ) \nonumber \\{} & {} - (r-\imath )^\upsilon (r-\imath +3+3 \upsilon ) \bigg ) \nonumber \\ \int _{t_\imath }^{t_{\imath +1}} (\theta -t_{\imath -2}) (\theta -t_{\imath -1}) (t_{r+1}-\theta )^{\upsilon -1} d \theta= & {} \frac{(\Delta t)^{\upsilon +2}}{\upsilon (\upsilon +1)(\upsilon +2)}\nonumber \\{} & {} \times \bigg [(r-\imath +1)^\upsilon V_1 -(r-\imath )^\upsilon V_2 \bigg ], \end{aligned}$$

where $V_1=2(r-\imath )^2+(3\upsilon +10) (r-\imath )+2\upsilon ^2+9 \upsilon +12,$ and $V_2=2(r-\imath )^2+(5\upsilon +10) (r-\imath )+6\upsilon ^2+18 \upsilon +12$. After simplification, we get that

26 $$\begin{aligned} g^{r+1}= & {} g^0+ \frac{1-\upsilon }{AB(\upsilon )} f (t_r,g(t_r)) \nonumber \\{} & {} +\frac{\upsilon (\Delta t)^\upsilon }{AB(\upsilon )\Gamma (\upsilon +1)} \sum ^{r}_{\imath =2} f (t_{\imath -2},g^{\imath -2}) [(r-\imath +1)^\upsilon - (r-\imath )^\upsilon ] \nonumber \\{} & {} +\frac{\upsilon (\Delta t)^\upsilon }{AB(\upsilon )\Gamma (\upsilon +2)} \sum ^{r}_{\imath =2} [f (t_{\imath -1},g^{\imath -1}) - f (t_{\imath -2},g^{\imath -2}) ] \nonumber \\{} & {} \times \bigg ( (r-\imath +1)^\upsilon (r-\imath +3+2 \upsilon ) - (r-\imath )^\upsilon (r-\imath +3+3 \upsilon ) \bigg ) \nonumber \\{} & {} +\frac{\upsilon (\Delta t)^\upsilon }{2AB(\upsilon )\Gamma (\upsilon +3)} \sum ^{r}_{\imath =2} [f (t_{\imath },g^{\imath })-2f (t_{\imath -1},g^{\imath -1}) + f (t_{\imath -2},g^{\imath -2}) ] \nonumber \\{} & {} \times \bigg [(r-\imath +1)^\upsilon V_1 -(r-\imath )^\upsilon V_2 \bigg ]. \end{aligned}$$

We will employ the aforementioned approach to depict the time series of the proposed infection model. Time series analysis holds significant importance in comprehending, monitoring, and managing diseases. It furnishes valuable insights into the dynamics of the disease, aids in the early detection of outbreaks, and enables the assessment of intervention effectiveness. This, in turn, contributes to more informed and targeted public health initiatives. The numerical values of system parameters and state variables will be assumed for computational purposes. Various simulations will be conducted to illustrate how these parameters impact the infection system.

In the initial simulation, illustrated in Figs. 2 and 3, we scrutinized the impact of the fractional parameter $\upsilon$ on the dynamics of HPV. In Fig. 2, we consider the values of $\upsilon$ to be 1.00, 0.95, 0.90,  and 0.85, while in Fig. 3, the value of $\upsilon$ is varied as 0.80, 0.70, 0.60,  and 0.50. This systematic exploration of diverse values for the input parameter $\upsilon$ allows us to thoroughly investigate the characteristic solution pathways of the system. The outcomes of these simulations unequivocally highlight the substantial influence exerted by the fractional parameter on the dynamics of the infection. Notably, $\upsilon$ emerges as a promising tool for effectively managing the spread of the infection within the community. Therefore, we strongly advocate for a more in-depth exploration and analysis of this fractional parameter by policymakers to enhance their understanding of its potential in mitigating the impact of the infection on public health. This comprehensive investigation can contribute valuable insights for developing targeted strategies in the control and prevention of the infection. Figure 4 depicts the impact of the input parameter $\beta$ on the dynamics of HPV infection. In this simulation, we considered $\beta$ values of 0.20, 0.40, 0.60, and 0.80. Our observations highlight the crucial role of this parameter, indicating a direct association with an increased risk of the infection.

In Figs. 5 and 6, we have illustrated the biological implications of varying input parameters $\rho$ and $\theta$ on the dynamics of HPV. In Fig. 5, we explored the effects of different values of $\rho$ (0.45, 0.55, 0.65, and 0.75), while maintaining $\theta$ at values of 0.2, 0.3, 0.4, and 0.5 in Fig. 6. Our investigation specifically focuses on discerning how changes in these parameters influence the behaviors of asymptomatic and infected individuals within the HPV system. In the conclusive simulation, depicted in Fig. 7, we investigated the impact of the input parameter $\eta$ on the solution pathways of HPV infection. For this analysis, we considered values of $\eta$ as 0.25, 0.30, 0.35, and 0.40. The observation centered on understanding how variations in $\eta$ contribute to the dynamics of the asymptomatic and infected classes within the model. These insights hold significant relevance for informing public health strategies, intervention measures, and the formulation of effective control policies aimed at managing and mitigating the repercussions of infectious diseases on populations. Understanding the intricate relationships between input parameters and the dynamics of HPV infection is essential for the development of targeted and efficient approaches to tackle such public health challenges.

## Conclusion

The infection HPV had posed a global public health challenge, especially in regions with limited access to health-care and preventive measures, contributing to health disparities and an increased disease burden. In our research, we structured a mathematical model for the transmission dynamics of HPV infection with the effect of vaccination, asymptomatic carrier and cervical cancer. We have shown that the solution of the recommended model are positive and bounded for positive initial values of state variables. We utilized the next-generation matrix method for the calculation of the basic reproduction number ${\mathcal {R}}_0$. In addition to this, we proved that the infection-free steady-state of the system are locally asymptotically stable for ${\mathcal {R}}_0<1$ and unstable in other cases. The existence of the solution has been investigated with the help of fixed-point theory. We introduced a numerical scheme to elucidate the dynamic behavior of the system, aiming to demonstrate the influence of the system’s input parameters. The most critical factors of the proposed system has been visualized and are recommended to the policy makers for the control and management of the infection. In the future research work, we will examine the impact of pulse vaccination on the dynamics of HPV infection. Additionally, we intend to incorporate the dynamics of HPV infection within a stochastic framework and conduct a comparative analysis of their respective outcomes.

## Ethical approval

There is no ethical issue in this work. All the authors actively participated in this research and approved it for publication.

## Data Availability

The datasets used and analysed during the current study available from the corresponding author on reasonable request.
